# LPS/Bcl3/YAP1 signaling promotes Sox9^+^HNF4α^+^ hepatocyte-mediated liver regeneration after hepatectomy

**DOI:** 10.1038/s41419-022-04715-x

**Published:** 2022-03-28

**Authors:** Changchun Shao, Yingying Jing, Shanmin Zhao, Xue Yang, Yiming Hu, Yan Meng, Yihua Huang, Fei Ye, Lu Gao, Wenting Liu, Dandan Sheng, Rong Li, Xiaoren Zhang, Lixin Wei

**Affiliations:** 1 Tumor Immunology and Gene Therapy Center, Third Affiliated Hospital of Second Military Medical University, Shanghai, 200438 China; 2 Institute of Translational Medicine, Shanghai University, Shanghai, 200444 China; 3 Laboratory Animal Center of Second Military Medical University, Shanghai, 200433 China; 4 Affiliated Cancer Hospital and Institute of Guangzhou Medical University, Guangzhou Municipal and Guangdong Provincial Key Laboratory of Protein Modification and Degradation, State Key Laboratory of Respiratory Disease, Guangzhou, 510000 China; 5 Department of Pathology, the School of Basic Medical Sciences, Fujian Medical University, Fuzhou, 350108 China

**Keywords:** Ubiquitylation, Transdifferentiation, NF-kappaB, Regeneration, Stem-cell research

## Abstract

Recent reports have demonstrated that Sox9^+^HNF4α^+^ hepatocytes are involved in liver regeneration after chronic liver injury; however, little is known about the origin of Sox9^+^HNF4α^+^ hepatocytes and the regulatory mechanism. Employing a combination of chimeric lineage tracing, immunofluorescence, and immunohistochemistry, we demonstrate that Sox9^+^HNF4α^+^ hepatocytes, generated by transition from mature hepatocytes, play an important role in the initial phase after partial hepatectomy (PHx). Additionally, knocking down the expression of Sox9 suppresses hepatocyte proliferation and blocks the recovery of lost hepatic tissue. In vitro and in vivo assays demonstrated that Bcl3, activated by LPS, promotes hepatocyte conversion and liver regeneration. Mechanistically, Bcl3 forms a complex with and deubiquitinates YAP1 and further induces YAP1 to translocate into the nucleus, resulting in Sox9 upregulation and mature hepatocyte conversion. We demonstrate that Bcl3 promotes Sox9^+^HNF4α^+^ hepatocytes to participate in liver regeneration, and might therefore be a potential target for enhancing regeneration after liver injury.

## Introduction

Liver regeneration is a fundamental pathophysiological process that occurs after the loss of hepatic tissue, and it is critical for prompting functional recovery and maintaining homeostasis. The molecular mechanisms involved in the regulation of liver regeneration are very intricate, and a deep understanding of the process will enable rational targeting with specific therapies to promote the recovery of liver function or inhibit the hepatocarcinogenesis caused by abnormal liver regeneration.

Following liver injury and partial surgical resection, the remaining mature hepatocytes replenish the lost tissue, restoring the liver to its full size and functionality [1]. In addition, when hepatocyte proliferation is seriously inhibited, hepatic progenitor cells (HPCs) participate in regeneration [2]. In contrast, there is evidence demonstrated that new hepatocytes do not originate from stem cells [3]. In addition, after extended injury, mature hepatocytes convert into progenitor-like cells, which possess bipotential characteristics and finally differentiate back into hepatocytes upon cessation of injury [4, 5]. Therefore, the cellular origin of liver regeneration is complex and requires further exploration.

In addition to the well-known hepatocytes, cholangiocytes, and HPCs, the hybrid hepatocytes (HybHPs) or Sox9^+^HNF4α^+^ hepatocytes were discovered recently [6]. These cells preexist in the periportal area and simultaneously express Sox9 and HNF4α. Additionally, HybHPs harbor high regenerative capacity and can repopulate the liver after chronic hepatocyte injury. As previously reported, mature hepatocytes harbor significant phenotypic plasticity and are transiently converted into HybHPs after liver injury [4, 7]. It is unclear whether HybHPs are derived from self-renewal, hepatocytes, or HPCs. In addition, the mechanisms involved in the regulation of HybHPs need further investigation.

Several studies have shown that innate immunity functions as an important stimulator of liver regeneration [8]. Among the different immunity pathways, the NF-κB pathway has a prominent role in the process of liver regeneration [9]. In rats, gut-derived LPS (lipopolysaccharide) levels in the portal and systemic plasma were much higher after PHx than in the control group [10]. LPS can activate the NF-κB pathway and promote hepatocyte proliferation [8]. Genome-wide binding analysis revealed that NF-κB dynamically regulated the differential expression of genes within multiple pathways after PHx [11]. Additionally, the NF-κB pathway is involved in phenotypic plasticity in several cell types, such as Th1 cells [12] and intestinal epithelial cells [13]. Whether the LPS/NF-κB pathway participates in the regulation of HybHP-mediated liver regeneration remains largely unclear.

The study aimed to determine the cell origin behind liver regeneration and the function of the LPS/NF-κB pathway to further uncover the molecular mechanism underlying this process. We performed PHx surgery to induce liver regeneration and demonstrated that Bcl3, which is activated by the LPS/NF-κB pathway, induced the conversion of mature hepatocytes into HybHPs and promoted hepatocyte proliferation. Notably, Bcl3 could bind with YAP1 and activate it by inhibiting its ubiquitination. The activation of YAP1 is vital for Bcl3-mediated liver regeneration. Our findings uncover a remarkable function of Bcl3 in controlling liver regeneration, which indicates that Bcl3 may be a potential therapeutic target in the promotion of liver regeneration and prevention of liver failure.

## Materials and methods

### Mouse strains

Six- to eight-week-old male C57BL/6 mice (weighing 20-22 g) were purchased from the Shanghai Experimental Animal Center of the Chinese Academy of Sciences (Shanghai, China). TLR4 knockout (*TLR4*^*−/−*^) mice were established by Nanjing Xunqi Biotechnology Co., Ltd. by CRISPR/Cas9-based genome editing. *Bcl3*^*-/-*^ mice were obtained from Prof. Xiaoren Zhang. *Fah*^−/−^ mice were from Prof. Yiping Hu. All animals were kept in a barrier room at an SPF animal facility in accordance with the Second Military Medical University Animal Care Committee.

### Hepatocyte transplantation and chimeric *Fah*^−*/−*^ mice

Parenchymal hepatocytes were isolated from wild-type C57BL/6 mice by a two-step collagenase perfusion, and then they were purified by a series of low-speed (1’ × 50*g*) centrifugation steps [14]. Cell number and viability were determined by trypan blue staining with a hemocytometer. Next, 5*10^5 hepatocytes were transplanted percutaneously into the spleens of *Fah*^*−/−*^ mice. Then, *Fah*^*−/−*^ mice were weaned from NTBC (2-(2-nitro-4-trifluoromethylbenzoyl)-1,3-cyclohexanedione) and maintained on water. Three weeks later, mutant mice were given NTBC for an additional 1 week, which was followed by drinking water for 3 weeks. The livers of *Fah*^−/−^ mice were allowed to repopulate for 10 weeks before PHx.

### 70% PHx

PHx was performed on mice as described previously [15]. At the indicated time points after PHx, experimental animals were sacrificed to remove the liver, and the liver-to-body-weight ratios were calculated. To evaluate the capacity for liver regeneration, the liver recovery ratio was calculated as follows: *R* = (*R*_3_ − *R*_0_)/*R*_0_ × 100%, where *R*_3_ and *R*_0_ represent the liver-to-body-weight ratio at day 3 and 0 after PHx, respectively. The liver tissues were fixed in formalin or frozen in OCT for cryosectioning, whereas the remaining tissues were stored at −80 °C until required.

### Cell lines

HEK293T cells were cultured in Dulbecco’s modified Eagle’s medium (DMEM, Gibco-BRL, Gaithersburg, USA) supplemented with 10% FBS, 1% penicillin, and 1% streptomycin in a humidified atmosphere with 5% CO_2_ at 37 °C. AML12 cells were cultured in DMEM/F12 containing 10% FBS, 5.5 mL of ITS Liquid Media Supplement (Sigma, USA), 40 ng/mL dexamethasone, 1% penicillin and 1% streptomycin. AML12 cells were treated with LPS (Sigma, Saint Louis, Missouri, USA) at 100 ng/ml at different times. All cell lines were purchased from ATCC.

### Gene silencing or overexpression mediated by virus gene transfer

We designed three shRNAs targeting Sox9 and YAP1, and a control shRNA serving as the negative control. pDKD-CMV-eGFP-U6-shRNA-Sox9 and pDKD-CMV-eGFP-U6-shRNA-YAP1 (Obio Technologies, Shanghai, China) were administered to mice randomly by tail vein injection. pAdeno-MCMV-Yap1-3Flag-IRES2-EGFP adenovirus overexpressed YAP1, and it was constructed by Obio Technologies. Three days after the virus injection, PHx was performed. The adeno-associated virus HBAAV2/9-CMV-M-Bcl3-3*flag-ZSGreen (Hanbio Biotechnology, Shanghai, China) was used to specifically overexpress Bcl3 in the liver. The virus was injected into the tail vein 2 weeks before PHx. Bcl3 shRNA plasmids were gifted from Dr. Xiaoren Zhang, and we cloned the Bcl3 shRNA sequences into the plasmid pLDK-CMV-EGFP-2A-Puro-U6-shRNA (Obio Technologies, Shanghai, China), a lentiviral vector. The lentivirus was packaged according to the manufacturer’s instructions. To obtain stable cell lines, lentivirus was added to AML12 cells at an MOI of 40, which was followed by screening with 1 µg/ml puromycin for 2 weeks. All target sequences are listed in Table S2.

### Plasmids

The plvx-IRES-puro-flag-mbcl3 plasmid was used to overexpress Bcl3, and it was obtained from Dr. Xiaoren Zhang. The YAP1 coding sequence was cloned into the plasmid pAdeno-MCMV-Yap1-HIS-IRES-EGFP (Obio Technologies, Shanghai, China). The pcDNA 3.1(+)-mUbb-myc plasmid was used to express myc-tagged Ubb protein, and it was constructed by GenScript (Shanghai, China).

### RNA extraction and real-time PCR

Total RNA was isolated from cell lines or tissues using an RNeasy^®^ Mini kit (Qiagen, Hilden, Germany) according to the manufacturer’s protocol. A Bestar^™^ qPCR RT Kit was used for reverse transcription (RT) to obtain cDNA, and real-time PCR was performed using an SYBR PrimeScript RT-PCR Kit (DBI, Ludwigshafen, Germany). The primer’s sequences are listed in Table S1. A mouse NF-kB Signaling Pathway PCR Array kit (Qiagen, Hilden, Germany) was used to analyze the differentially expressed genes after PHx. Data analysis was conducted at Qiagen’s GeneGlobe Data Analysis Center using a software-based tool.

### Western blotting analysis

Tissues or cultured cells were lysed with RIPA lysis buffer with protease inhibitors and phosphatase inhibitors (Beyotime, Shanghai, China) using standard methods. After centrifugation and quantification, 25 µg of protein was loaded for blotting with the indicated specific antibodies. A NE-PER Nuclear and Cytoplasmic Extraction kit (Thermo Fisher Scientific, Waltham, USA) was used to isolate nuclear and cytoplasmic fractions according to the manufacturer’s protocol. The following antibodies were used: anti-Sox9 (1:1000, Abcam, Cambridge, UK), anti-HNF4α (1:2000, Abcam, Cambridge, UK), anti-TERT (1:1000, Novus, Colorado, USA), anti-E-Cadherin (1:1000, Abcam, Cambridge, UK), anti-Vimentin (1:1000, CST, Danvers, USA), anti-CK19 (1:1000, Abcam, Cambridge, UK), anti-LGR5 (1:1000, Abcam, Cambridge, UK), anti-Epcam (1:1000, Abcam, Cambridge, UK), anti-P65 (1:1000, CST, Danvers, USA), anti-p-P65 (1:1000, CST, Danvers, USA), anti-Bcl3 (1:100, Abcam, Cambridge, UK), anti-Histone H3 (1:1000, Proteintech, Rosemont, USA), anti-YAP1 (1:1000, CST, Danvers, USA), anti-CTGF (1:1000, Abcam, Cambridge, UK), anti-Cyr61 (1:1000, Abcam, Cambridge, UK), anti-His (1:5000, Proteintech, Rosemont, USA), anti-Flag (1:2000, Proteintech, Rosemont, USA), anti-Myc (1:1000, Proteintech, Rosemont, USA), anti-Ubiquitin (1:1000, CST, Danvers, USA), and anti-β-actin (1:4000, Bioworld, MN, USA).

### Immunoprecipitation

Before harvesting them, HEK293T cells were transfected with the indicated plasmids using Lipofectamine 3000 (Invitrogen, Carlsbad, CA). Primary hepatocytes were purified at the indicated times after PHx. Cells or primary hepatocytes were lysed in IP lysis/wash buffer supplemented with phosphatase and protease inhibitors. The total protein concentration of the supernatants was quantified using a BCA Protein Assay kit (Beyotime, Shanghai, China) after centrifugation for 10 min at 13,000 × *g* at 4 °C. One milligram of total protein was mixed with 2 μg of the indicated primary antibody or isotype control IgG, and the mixture was shaken on a rotating shaker at 4 °C overnight. Immunoprecipitates were collected and washed three times with lysis buffer, and proteins were analyzed by western blot.

### Ubiquitination assay

HEK293T cells were transiently transfected with His-YAP1, Flag-Bcl3, and Myc-Ub. Forty-eight hours later, the cells were treated with 20 µM MG132 for 8 h before harvesting. Lysed samples were immunoprecipitated with a His antibody (1:50, Proteintech, Rosemont, USA) using a Pierce™ Classic IP Kit (Thermo Fisher Scientific, Waltham, USA) according to the manufacturer’s protocols. Finally, an anti-Myc antibody was used to detect Ub conjugates by western blot analysis.

### ChIP assay

A ChIP Assay kit (Beyotime, Shanghai, China) was used to perform the ChIP assay according to the manufacturer’s instructions [16]. Bcl3 binding sites in the *Sox9* promoter region (approximately 2 kb upstream of the coding region) were predicted by JASPAR 2020 (http://jaspar.genereg.net/). Briefly, AML12 cells were collected and fixed with 1% formaldehyde for 10 min at 37 °C, and then chromatin was shredded to fragments of 200–1000 bp by sonication. The sonicated DNA fragments were incubated with antibodies against Bcl3 (1:50, Abcam, Cambridge, UK) or used in a control incubation overnight at 4 °C. Immunocomplexes were washed and purified using a DNeasy Blood & Tissue kit (Qiagen, Hilden, Germany). Enriched DNA was measured using qPCR. The primers are listed in Table S1.

### Immunohistochemistry analysis

Paraffin-embedded liver samples were cut into sections for hematoxylin–eosin and immunohistochemistry staining, and the procedures were performed as previously described [17]. The following antibodies were used in the IHC analysis: anti-Sox9 (1:200, Abcam, Cambridge, UK), anti-HNF4α (1:2000, Abcam, Cambridge, UK), anti-glutamine synthetase (1:2000, BD, New Jersey, USA), anti-Ki67 (1:100, Abcam, Cambridge, UK), anti-PCNA (1:1000, Proteintech, Rosemont, USA), anti-E-cadherin (1:200, Abcam, Cambridge, UK), anti-Vimentin (1:200, CST, Danvers, USA), and anti-YAP1 (1:200, CST, Danvers, USA). At least three random areas per slide were selected to count the number of positively stained cells.

### Immunofluorescence analysis

Liver tissues were fixed in 4% paraformaldehyde overnight and then were cryopreserved in 30% sucrose solution before freezing in OCT tissue blocks. Blocked tissues were cut into 8 μm serial sections for immunofluorescence staining [4]. Approximately, 1 × 10^4^ AML12 cells were seeded in a 48-well dish and then were fixed in 4% paraformaldehyde for immunofluorescence staining after 12 h. The detailed method has been published previously [17]. For triple-label immunofluorescence, after routine dewaxing and hydration, antigen retrieval was carried out in EDTA buffer (pH 8.0) using a microwave antigen repair technique. Slides were incubated in blocking buffer (0.5% bovine serum albumin in PBS) for 30 min at room temperature, which was followed by incubation with the first primary antibody at 4 °C overnight. The next day, sections were incubated with an horseradish peroxidase (HRP)-labeled secondary antibody for 50 min at room temperature. After washing, slides were incubated with Cy3-labeled tyramides for 10 min at room temperature in the dark. After washing in PBS-0.1% Tween, slides were incubated in EDTA buffer (pH 8.0) using a microwave antigen retrieval technique to remove the primary and secondary antibodies that have been incorporated into the tissues. Then, slides were incubated in the next primary antibody at 4 °C overnight, followed by HRP-labeled secondary antibody, then by FITC-labeled tyramides. The third primary antibody was labeled as described before, and the second antibody was replaced by a Cy5-labeled secondary antibody for 50 min at room temperature in the dark. Finally, slides were incubated with DAPI for nuclear staining. Imaging was performed with Pannoramic Viewer (Budapest, Hungary). The antibodies used were as follows: anti-Sox9 (1:200, Novus, Colorado, USA), anti-HNF4α (1:2000, Abcam, Cambridge, UK), anti-Fah (1:200, ABclonal, China), anti-YAP1 (1:200, Santa Cruz Biotechnology, USA), anti-Bcl3 (1:50, Abcam, Cambridge, UK), anti-TERT (1:100, Novus, Colorado, USA), anti-CK19 (1:200, Abcam, Cambridge, UK), and anti-LGR5 (1:100, Abcam, Cambridge, UK).

### Measurement of plasma LPS

Three hours after PHx, portal vein blood samples were collected into sterile tubes to obtain plasma. The levels of hepatic portal vein LPS were detected by a Bioendo^TM^ EC Endotoxin Test kit (End-point Chromogenic Assay, Diazo Coupling) (Xiamen Bioendo Technology, Xiamen, China) according to the manufacturer’s protocols.

### Statistical analysis

The results are expressed as the mean ± SEM. The unpaired two-tailed Student’s *t*-test was used to evaluate statistical significance by GraphPad Prism 7 (GraphPad Software Inc., San Diego, CA). All experiments and analysis were conducted in investigator blinded fashion. All experiments were performed at least three times. *P* values of less than 0.05 were considered statistically significant for all statistical analyses.

## Results

### HybHPs increase in the priming phase after PHx

**Fig. 1:**
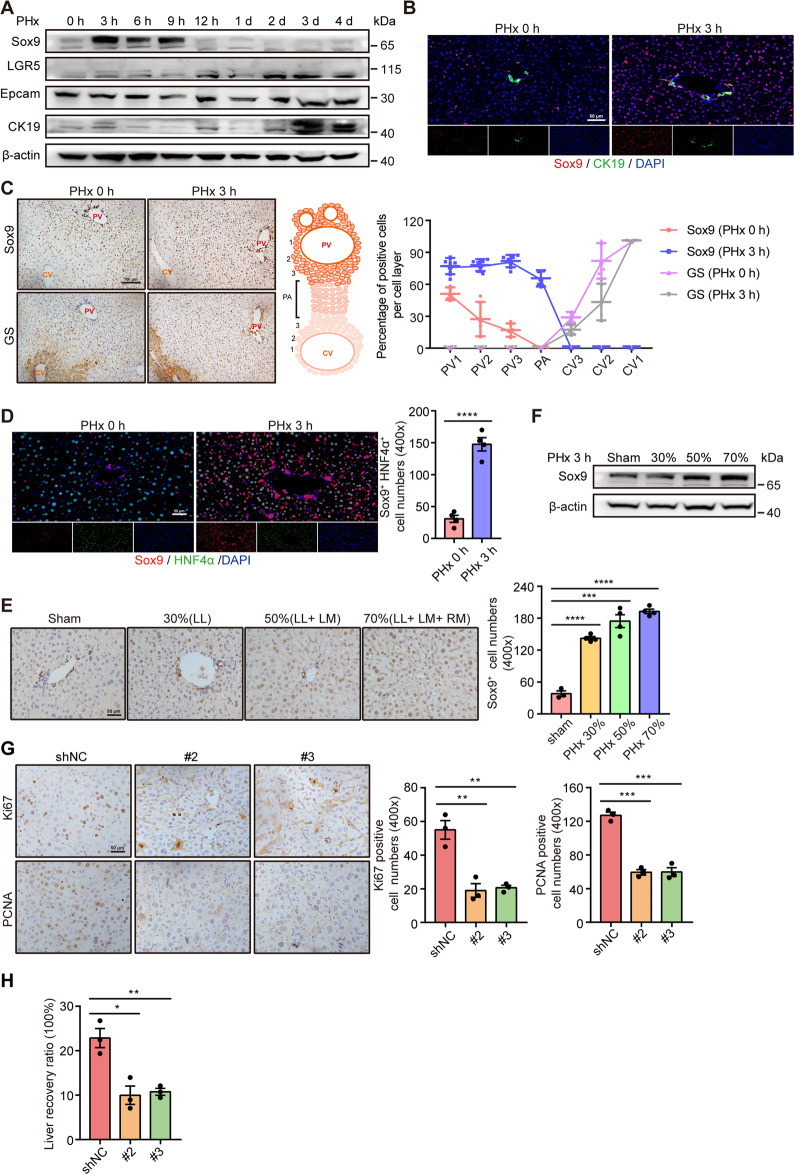
Sox9^+^HNF4α^+^ hepatocytes are involved in liver regeneration after PHx. **A** Representative western blotting analysis of liver stem cell markers at the indicated times after PHx. **B** Representative triple staining for Sox9 (red) and CK19 (blue) in the liver after PHx in wild-type mice. **C** Left: IHC analysis of liver at the indicated times after PHx to determine Sox9 and GS expression. GS functions as a maker for the central vein area. Right: Percentage of hepatocytes expressing GS and Sox9 in three cell layers around the central and portal veins and in the parenchyma (as indicated in the schematic). **D** Sox9 (red) and HNF4α (green) double staining of liver sections at the indicated times after PHx. (Left panel, representative images; right panel, quantitative analysis of Sox9^+^HNF4α^+^ cell numbers). **E** Left: IHC analysis of Sox9 in livers at 3 h after different proportions were removed by PHx. Right: Quantification of Sox9^+^ cell numbers. **F** The relative expression of Sox9 protein after different proportions of the liver were removed by hepatectomy. **G** Left: Relative expression of Ki67 and PCNA revealed by IHC staining in pDKD-Control and pDKD-Sox9 shRNA mice at the indicated times after PHx. Right: Quantification of Ki67- or PCNA-positive cells. **H** Liver recovery ratio in pDKD-Control and pDKD-Sox9 shRNA mice at day 3 after PHx. The data are expressed as the mean ± SEM. The measurement results were repeated at least 3 times and the results were similar. *p < 0.05, **p < 0.01, ***p < 0.001, and ****p < 0.0001.

To try and address whether liver stem cells participated in liver regeneration after PHx, we carried out 70% PHx in mice [15], and then examined the expression of the liver stem cell markers Sox9, LGR5, Epcam, and CK19 (Fig. 1A). The data showed that Sox9 expression was much higher at 3 h after PHx, and then it gradually decreased to the normal level. For Epcam and LGR5, there were no major changes. CK19 was only increased at 3 days post-PHx. We found that Sox9 and CK19 were co-located in the bile duct cells before PHx, and cells around the bile duct were Sox9^+^ at 3 h post PHx (Fig. 1B). Serial sections revealed that Sox9^+^ cells were located in the periportal area, away from the GS^+^ (glutamine synthetase) area (Fig. 1C), and these Sox9^+^ cells were similar to HybHPs reported previously [6]. To illustrate whether the Sox9^+^ cells were HybHPs, we performed Sox9 and HNF4α double staining and found that these increased Sox9^+^ cells were positive for HNF4α (Fig. 1D). Thus, our results indicated that these Sox9^+^ cells were HybHPs. Next, we found that the number of Sox9^+^ cells was much higher when a larger proportion of the liver was removed (Fig. 1E). Immunoblot analyses also confirmed higher levels of Sox9 protein (Fig. 1F). Our findings showed that HybHPs appeared quickly after PHx; thus, we surmised that they might play an important role in regeneration after PHx.

TERT^High^ hepatocytes, located in the periportal region, can repopulate the liver for homeostasis [18]. Thus, we performed an immunofluorescence assay to examine the expression of TERT in HybHPs (Fig. S1A). The data showed that Sox9, HNF4α, and TERT were colocalized. Moreover, TERT was gradually increased after PHx, and induced when increasing proportions of the liver were removed (Fig. S1B). During tissue repair, mature somatic cells exhibit stem-like properties after induction into the EMT program [19]. Consistent with this, IHC and Western blot assay indicated that Vimentin expression was much higher after PHx (Fig. S1C, D). All these data indicate that HybHPs possess high stemness and proliferation potential.

To extend these observations, we administered wild-type C57BL/6 mice with an adenovirus to silence Sox9. Knockdown Sox9 decreased the number of Ki67^+^ and PCNA^+^ cells and reduced the liver recovery ratio 3 days after PHx (Fig. S1E and Fig. 1G, H). Thus, our data demonstrated that Sox9 acts as a functional molecule to participate in liver regeneration.

Our data showed that HybHPs were increased after PHx, but the origin of these cells remained unclear. They may originate from self-duplication, HPCs or mature hepatocytes. Serial sections revealed that the number of Sox9^+^ cells was highly increased at 3 h after PHx, but very few Ki67^+^ proliferating cells were found (Fig. S1F). As shown in Fig. 1A, there were no obvious differences for other HPC makers, which indicates that HPCs were not activated. A previous report indicated that HybHPs, participating in PCN-induced liver enlargement, were of hepatocyte origin [20]. To further strengthen these observations, we transplanted Fah^+^ hepatocytes into *Fah*^*−/−*^ mice and tracked the fate of Fah^+^ hepatocytes after PHx. Ten weeks post transplantation, IF analysis confirmed that Fah was widely expressed in the chimeric livers (Fig. S1G). Then, we performed PHx operations and found that some of Sox9^+^ cells were colocalized with Fah (Fig. S1H). Overall, our results indicate that mature hepatocyte can give rise to HybHPs after PHx.

### LPS/TLR4 pathway activation induces hepatocyte transition after PHx

**Fig. 2:**
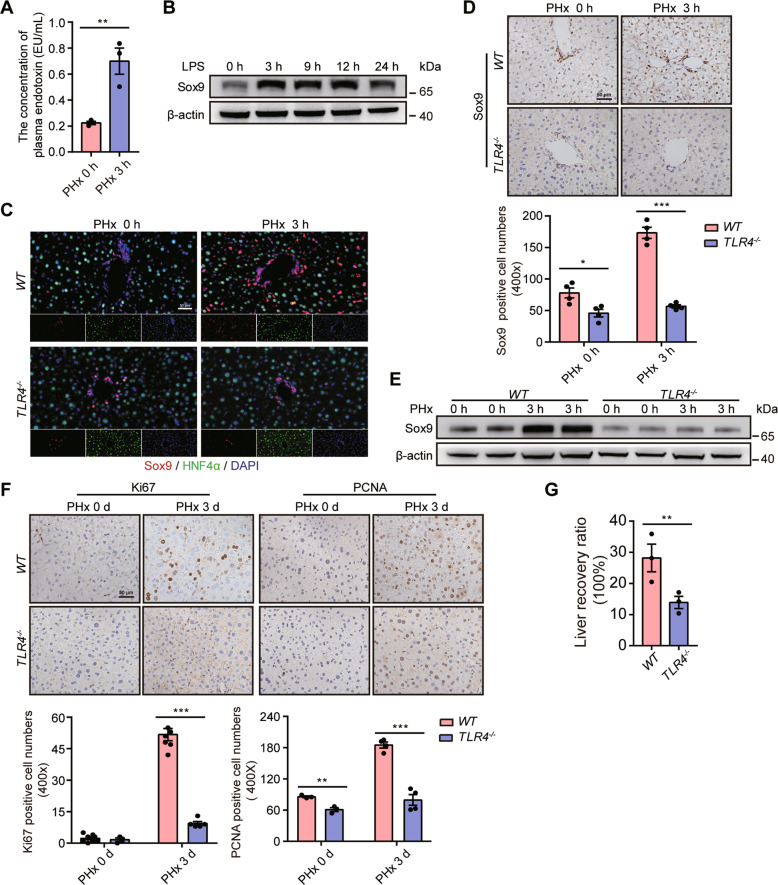
The LPS/TLR4 signaling pathway induces the transition of hepatocytes into Sox9^+^HNF4α^+^ cells and promotes liver regeneration. (**A**) The levels of plasma LPS from the portal vein in the control and PHx groups at 3 h post-surgery. (**B**) The relative expression of Sox9 was assessed by western blotting in AML12 cells after stimulation with LPS for the indicated times. (**C**) Double staining of liver sections for Sox9 (red) and HNF4α (green) at hours 0 and 3 after PHx in wild-type and *TLR4*^*-/-*^ mice. (**D**) Upper: Representative Sox9 staining in wild-type and *TLR4*^*-/-*^ mice after PHx. Bottom: Quantification of Sox9-positive cell numbers. (**E**) Representative western blotting analysis of Sox9 in wild-type and *TLR4*^*-/-*^ mice after PHx. (**F**) Upper: IHC analysis of Ki67 and PCNA in liver tissue after PHx in wild-type and *TLR4*^*-/-*^ mice. Bottom: Quantification of Ki67- or PCNA-positive cells. (**G**) Liver recovery ratio in wild-type and *TLR4*^*-/-*^ mice at 3 days after PHx. The data are expressed as the mean ± SEM. The measurement results were repeated at least 3 times and the results were similar. *p < 0.05, **p < 0.01, and ***p < 0.001.

We next investigated the mechanism underlying the phenomenon. LPS levels are increased after PHx, and exogenous LPS administration before PHx accelerates hepatic DNA synthesis [10]. Thus, we speculated that LPS might induce the transition of mature hepatocytes post PHx. To explore this, we first examined LPS levels after PHx and found that it was much higher in the PHx group than in the control group (Fig. 2A). Next, we found that LPS could upregulate Sox9 expression in AML12 cells (Fig. S2A and Fig. 2B). In addition, IF assays indicated that Sox9 was strikingly enriched in the nucleus (Fig. S2B). Next, qRT-PCR assays showed that the stemness markers and some pro-EMT transcription factors were significantly upregulated (Fig. S2C, D). Furthermore, TERT expression was also upregulated after induction by LPS (Fig. S2D). Additionally, E-cadherin expression gradually decreased and Vimentin expression subsequently increased.

The portal vein carries blood from the gastrointestinal tract, which contains abundant LPS. Therefore, we performed an operation where we ligated the left hepatic portal vein branch to evaluate the function of LPS. The result showed that Sox9 was much higher in the hepatic lobe with no ligation than it was in the lobe connected to the ligated portal vein (Fig. S2E, F). These data revealed that LPS might participate in the conversion of mature hepatocytes into HybHPs.

Previous data have shown that LPS binds to TLR4 to regulate liver regeneration after PHx [21]. To further strengthen these hypotheses, we performed PHx operations in *TLR4*^*-/-*^ mice. Compared with wild-type mice, enrichment of HybHPs was not observed in *TLR4*^−*/*−^ mice (Fig. 2C). Similarly, IHC and western blot analysis confirmed that Sox9 expression was not induced in *TLR4*^*−/−*^ mice (Fig. 2D, E). TLR4 deficiency significantly inhibited the increase in the number of Ki67^+^ and PCNA^+^ hepatocytes (Fig. 2F). Accordingly, recovery of the liver-to-body-weight ratio was markedly delayed in *TLR4*^*−/−*^ mice (Fig. 2G). Altogether, these data highlight that activation of LPS/TLR4 signaling induces hepatocyte transition and promotes liver regeneration after PHx.

### Bcl3, as a master downstream molecule of the LPS/TLR4 pathway, participates in liver regeneration

**Fig. 3:**
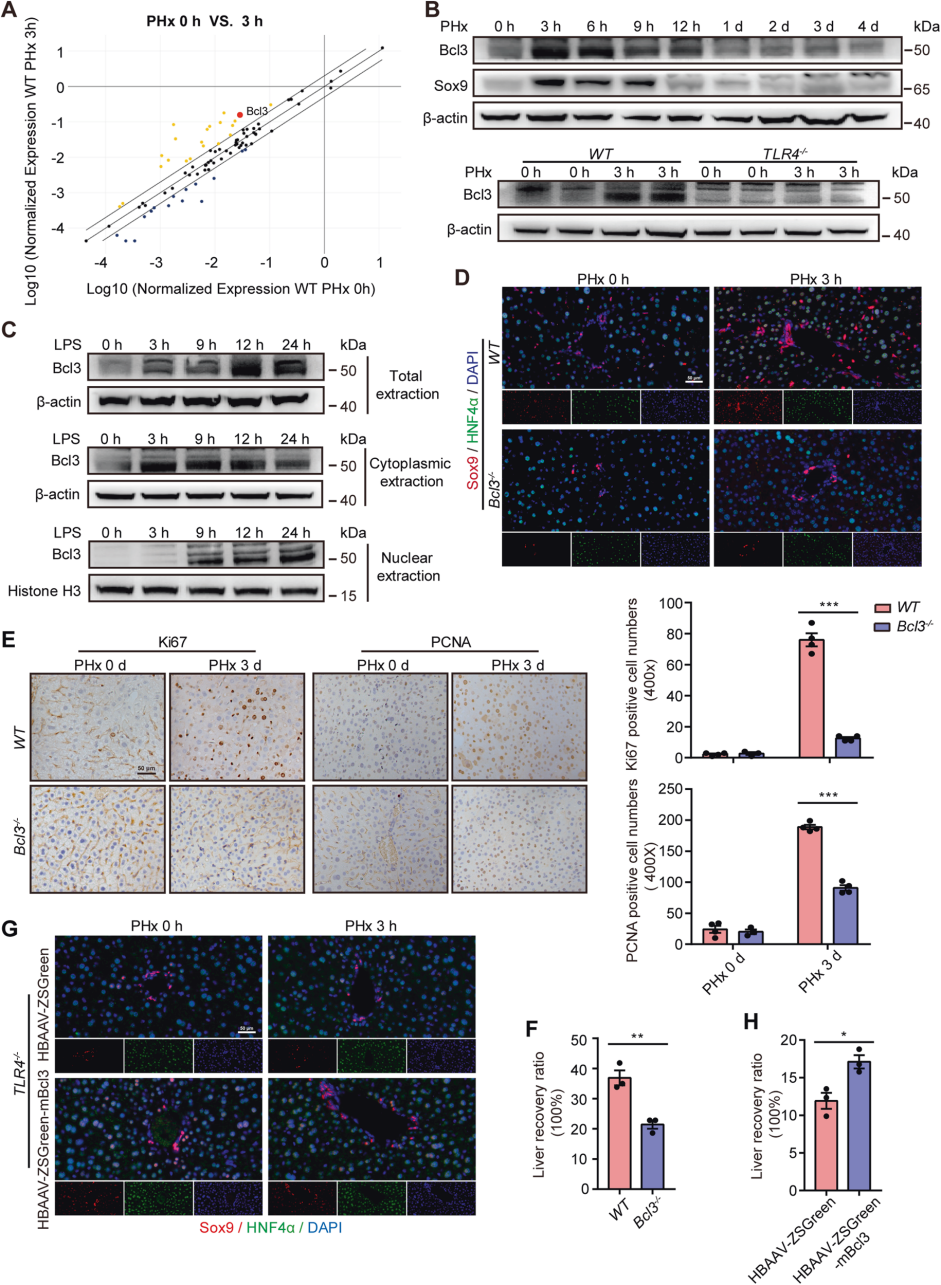
Bcl3 activation by the LPS/TLR4 signaling pathway is involved in liver regeneration after PHx. **A** Differentially expressed genes in the initial phase after PHx determined by PCR array analysis of the NF-κB signaling pathway. **B** Upper: Representative images showing Bcl3 and Sox9 levels, as assessed by western blotting of the liver at the indicated times after PHx. Bottom: Representative image showing Bcl3 levels, as assessed by western blotting in wild-type and *TLR4*^*−/−*^ mice at the indicated times after PHx. **C** Western blotting analysis of Bcl3 in total, cytoplasmic and nuclear extracts from AML12 cells at the indicated times after treatment with LPS. **D** Double staining of Sox9 (red) and HNF4α (green) in liver sections at hours 0 and 3 after PHx in wild-type and *Bcl3*^−*/−*^ mice. **E** Left: IHC analysis of Ki67 and PCNA in liver sections from wild-type and *Bcl3*^−/−^ mice at days 0 and 3 after PHx. Right: Quantification of Ki67- or PCNA-positive cells. **F** Liver recovery ratio in wild-type and *Bcl3*^−/−^ mice. **G** Two weeks after administration of HBAAV-ZSGreen or HBAAV-ZSGreen-mBcl3 virus, TLR4-deficient mice were subjected to PHx, and IF analysis of Sox9 (red) and HNF4α (green) was performed at the indicated times. **H** Liver recovery ratio in *TLR4*^*−/−*^ mice after administration of HBAAV-ZSGreen or HBAAV-ZSGreen-mBcl3 virus. The data are expressed as the mean ± SEM. The measurement results were repeated at least 3 times and the results were similar. *p < 0.05, **p < 0.01, and ***p < 0.001.

To investigate the mechanism, we performed a PCR array analysis of the NF-κB pathway (Fig. 3A), which is an important downstream pathway of LPS/TLR4 signaling [8]. Among the top ten differentially expressed genes, Bcl3 encodes a transcription coactivator, which is correlated with stemness, EMT and proliferation [22–24]. Western blot analysis revealed that Bcl3 was significantly increased post PHx and then it gradually recovered to normal levels, and *TLR4* knockout inhibited the upregulation (Fig. 3B). These results suggest that Bcl3 may participate in liver regeneration after PHx.

Next, we examined Bcl3 expression in AML12 cells after stimulation with LPS and found that LPS could upregulate Bcl3 (Fig. S3A and Fig. 3C). IF and western blot assays indicated that the increased Bcl3 was mainly located in the nucleus (Fig. S3B and Fig. 3C). Likewise, Bcl3 was highly enriched in the unligated hepatic lobe of PHx mice (Fig. S3C). Overall, these results reveal that Bcl3, which is induced by the LPS/TLR4 pathway, might participate in the process of regeneration.

Next, we performed PHx in *Bcl3*^*−/−*^ mice and found that very few cells coexpressed Sox9 and HNF4α (Fig. 3D). Consistently, upregulation of Sox9 was detected in wild-type mice at 3 h after PHx but not in *Bcl3*^*−/−*^ mice (Fig. S3D, E). Furthermore, Bcl3 deficiency reduced the proliferation of hepatocytes and the liver-to-body-weight ratio (Fig. 3E, F).

Our data demonstrated that Bcl3 was activated after PHx, and Bcl3 deficiency inhibited liver regeneration. We then asked whether Bcl3 could rescue the inhibited liver regeneration in *TLR4*^*−/−*^ mice. We overexpressed Bcl3 in *TLR4*^*−/−*^ mice and found that there were many more HybHPs in *TLR4*^*−/−*^ mice (Fig. 3G). Also, Bcl3 overexpression in *TLR4*^*−/−*^ mice was able to rescue Sox9 expression post-PHx (Fig. S3F, G). As predicted, the number of PCNA^+^ hepatocytes and the liver-to-body-weight ratio in the Bcl3-overexpressing group was much higher than it was in the control group (Figs. S3H and 3H). Taken together, our results revealed that Bcl3, acting as a master downstream molecule of the LPS/TLR4 pathway, promotes liver regeneration after PHx.

### Bcl3-induced liver regeneration is mediated by YAP1 activation

**Fig. 4:**
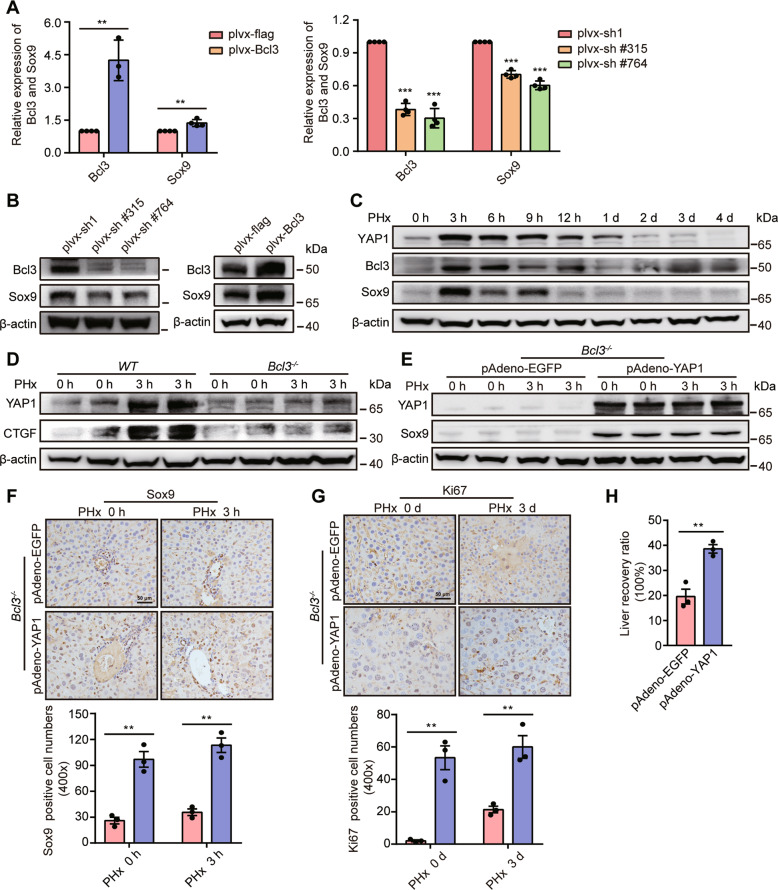
YAP1 functions as a downstream target of Bcl3 to participate in liver regeneration. **A**, **B** qRT-PCR (**A**) and western blotting analysis (**B**) of Bcl3 and Sox9 in AML12 cells after Bcl3 overexpression or silencing. **C** Representative images showing YAP1, Bcl3, and Sox9 levels, as assessed by western blotting of the liver at the indicated times after PHx. **D** Representative western blotting analysis of YAP1 and CTGF in wild-type and Bcl3-deficient mice at the indicated times after PHx. **E** The relative expression of YAP1 and Sox9 in Bcl3-deficient mice treated with pAdeno-EGFP or pAdeno-YAP1 virus. **F**, **G** Representative IHC analysis of Sox9 (**F**) and Ki67 (**G**) in Bcl3-deficient mice after YAP1 overexpression. **H** Liver recovery ratio in *Bcl3*^*−/*^^−^ mice treated with pAdeno-EGFP or pAdeno-YAP1 virus. The data are expressed as the mean ± SEM. The measurement results were repeated at least 3 times and the results were similar. **p < 0.01, and ***p < 0.001.

We next investigated how Bcl3 regulates the conversion of hepatocytes after PHx. We found that Sox9 was highly enriched in primary hepatocyte lysates from wild-type mice 3 h post PHx, but not in Bcl3-deficient mice (Fig. S4A). And Bcl3 positively regulated Sox9 expression in AML12 cells (Fig. 4A, B), which indicates that the regulation was mainly at the transcriptional level. Bcl3 forms a complex with p50 or p52 homodimers to regulate the transcription of NF-κB target genes [23]. Therefore, we analyzed the promoter of the *Sox9* gene and found that it contained three potential κB binding sites (Fig. S4B). However, chromatin immunoprecipitation (ChIP) assay revealed that Bcl3 could not bind to these sites (Fig. S4C). This indicates that Bcl3 might not directly bind to the promoter of *Sox9* by combining with NF-κB.

The Hippo/YAP signaling pathway plays a vital role in the liver, and YAP1 can regulate the transcription of *Sox9* [5]. Therefore, we hypothesized that YAP1 mediated the pro-regeneration effects of Bcl3. IF assays for Sox9 and YAP1 indicated that the broadly expressed Sox9 was colocalized with YAP1 after PHx (Fig. S4D). YAP1 was increased and then gradually decreased to normal levels, consistent with the expression patterns of Bcl3 and Sox9 (Fig. 4C). In addition, Bcl3 deficiency inhibited the upregulation of YAP1 and CTGF after PHx (Fig. 4D and Fig. S4E). And YAP1 knockdown inhibited Sox9 and TERT expression in wild-type mice after PHx (Fig. S4F). As expected, YAP1 silence decreased the number of HybHPs and Ki67^+^ cells, and the recovery of the liver-to-body-weight ratio (Fig. S4G–I).

Next, we cultured AML12 cells with LPS to examine the expression of YAP1. After stimulation, *YAP1* mRNA was not significantly different, but *CTGF* and *Cyr61* mRNAs were strongly induced (Fig. S5A). However, the protein expression of *YAP1*, *CTGF*, and *Cyr61* was increased (Fig. S5B). Also, we found that increased YAP1 was mainly located in the nucleus (Fig. S5C). Furthermore, results from the hepatic lobe ligation model indicated that LPS also activated YAP1 in vivo (Fig S5D). These data revealed that Bcl3-induced liver regeneration might be mediated by YAP1 activation.

To further confirm that YAP1 acts as a downstream molecule of Bcl3 to participate in liver regeneration, we overexpressed YAP1 in *Bcl3*^*−/−*^ and *TLR4*^−*/−*^ mice. The results demonstrated that YAP1 overexpression rescued the decreased expression of Sox9, upregulated Ki67 expression, and promoted recovery of the liver-to-body-weight ratio post-PHx in *Bcl3*^−*/−*^ mice (Fig. 4E–H). In *TLR4*^−*/−*^ mice, the increase in YAP1 and CTGF after PHx was inhibited compared to controls (Fig. S6A). After YAP1 overexpression in *TLR4*^−*/−*^ mice, Sox9 expression, and the number of HybHPs and PCNA^+^ hepatocytes were increased (Fig S6B-E). Also, recovery of the liver-to-body-weight ratio was enhanced (Fig. S6F). Overall, our results support the idea that LPS promotes liver regeneration by activation of Bcl3/YAP1.

### Bcl3 binds to and deubiquitinates YAP1

**Fig. 5:**
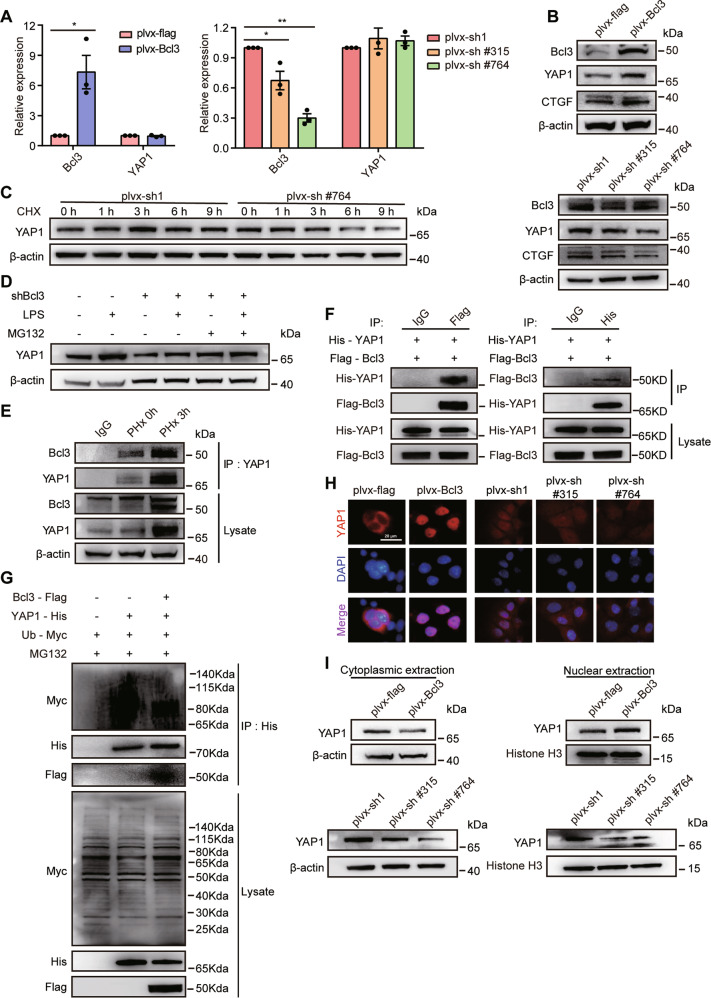
Bcl3 interacts with and stabilizes YAP1 by inhibiting its ubiquitination. **A** The relative expression of *YAP1* at the RNA level after Bcl3 was silenced or overexpressed in AML12 cells. **B** Western blotting analysis of Bcl3, YAP1, and its target CTGF after Bcl3 was silenced or overexpressed in AML12 cells. **C** Forty-eight hours after infection with the indicated shRNA lentivirus, AML12 cells treated with 20 µg/ml CHX at the indicated times were collected and analyzed for YAP1 expression at the protein level. **D** After infection with control or Bcl3 shRNA virus for 48 h, AML12 cells were treated with 100 ng/ml LPS for 12 h, then with 20 µM MG132 for 8 h. Cells were subsequently harvested to analyze YAP1 expression by western blotting. **E** Primary hepatocytes in wild-type mice were isolated at the indicated times after PHx, and cell lysates were immunoprecipitated with a YAP1 antibody. Western blotting was used to analyze the level of Bcl3 in the immunocomplexes. **F** HEK293T cells were transfected with His-YAP1 and Flag-Bcl3 plasmids for 48 h. After that, cell lysates were immunoprecipitated with the indicated antibodies. The immunocomplexes were subjected to western blotting using anti-His or anti-Flag antibodies. **G** HEK293T cells were transfected with the indicated plasmids for 48 h and then treated with 20 µM MG132 for 8 h. Cells were harvested and subjected to immunoprecipitation by His antibody. Then, immunocomplexes were analyzed by western blotting with the indicated antibodies. **H** IF was used to analyze the expression of YAP1 (red) in AML12 cells after Bcl3 silencing or overexpression. **I** Western blotting analysis with YAP1 antibody using cytoplasmic and nuclear extracts from AML12 cells after Bcl3 silencing or overexpression. Histone H3 was used as a nuclear loading control. The data are expressed as the mean ± SEM. The measurement results were repeated at least three times and the results were similar. *p < 0.05, and **p < 0.01.

To further clarify the molecular basis of Bcl3 knockdown-mediated YAP1 downregulation, we performed additional assays. We found that Bcl3 overexpression or knockdown in AML12 cells did not affect *YAP1* mRNA levels (Fig. 5A), but regulated *YAP1* protein levels positively (Fig. 5B). Previous studies showed that Bcl3 can stabilize a p50 complex by blocking the ubiquitination of p50 [25]. We hypothesized that Bcl3 might regulate YAP1 stability. Therefore, we analyzed the half-life of YAP1 after Bcl3 silencing and found that YAP1 protein was less stable (Fig. 5C). In addition, LPS administration upregulated YAP1, which was inhibited after Bcl3 knockdown; however, MG132 partially restored the downregulation of YAP1 after Bcl3 silencing (Fig. 5D). Taken together, these results indicate that Bcl3 might regulate YAP1 stability in protein levels.

To further investigate whether Bcl3 stabilizes YAP1 by regulating its ubiquitination, we performed coimmunoprecipitation experiments. We found that endogenous Bcl3 was coimmunoprecipitated with endogenous YAP1 in primary hepatocytes, and this interaction was much stronger after PHx (Fig. 5E). Coimmunoprecipitation assays also demonstrated that exogenous Bcl3 and YAP1 can interact with each other in HEK293T cells (Fig. 5F). A ubiquitination assay showed that overexpressed Bcl3 was able to decrease YAP1 ubiquitination (Fig. 5G). Overall, these results suggest that Bcl3 stabilizes YAP1 by directly interacting with it and inhibiting its ubiquitination.

To further examine whether Bcl3 regulates the subcellular location of YAP1, we performed an IF assay and found that Bcl3 could induce the translocation of YAP1 into the nucleus (Fig. 5H). The western blotting analysis also confirmed this phenomenon (Fig. 5I). These results reveal that Bcl3 stabilizes YAP1 and promotes the nuclear translocation of YAP1.

**Fig. 6:**
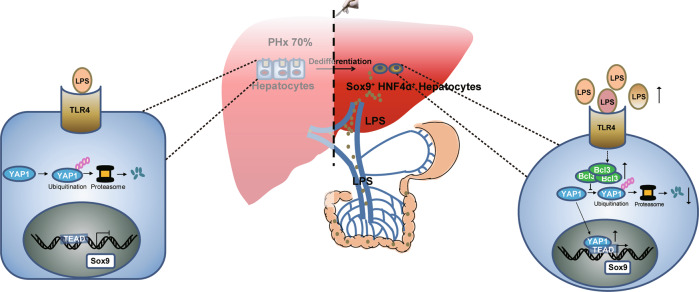
Schematic representation of liver regeneration mediated by the LPS/Bcl3-YAP1 axis. LPS, derived from enteric bacteria, promotes liver regeneration by inducing the conversion of mature hepatocytes into Sox9^+^HNF4α^+^ cells, which is mediated by the activation of Bcl3 and YAP1.

Altogether, these data provide evidence that when Bcl3 is induced by the activated LPS/TLR4 pathway, it promotes the conversion of mature hepatocytes into HybHPs by YAP1 activation to promote liver regeneration after PHx (Fig. 6).

## Discussion

Previous reports reveal that liver regeneration after PHx is only dependent on the proliferation of the mature cellular population and is not reliant on HPCs [1, 2, 26]. There is no evidence demonstrating that stem cells participate in the process. Here, we found that HybHPs are greatly expanded and play a vital role in the PHx model. This finding is consistent with a previous report that HybHPs are located at the periportal region, which is an organizing center for liver repair with a functional stem cell niche [27].

Our results revealed that mature hepatocytes have significant phenotypic plasticity in liver regeneration after PHx. The conversion of mature hepatocytes into progenitor-like cells is a general phenomenon that often occurs in chronic liver injury, particularly in response to biliary damage [4, 28]. We are the first to demonstrate that the conversion of hepatocytes into HybHPs is significantly increased after PHx. In addition, during the process of conversion, hepatocytes acquire mesenchymal cell features, as indicated by increased expression of the mesenchymal cell marker Vimentin. This is consistent with a previous report that the transition of hepatocytes into hepatocyte-derived progenitor cells is correlated with the induction of mesenchymal genes in the injured liver [4]. Previous data indicated that HybHPs appear upon liver injuries or PXR-induced hepatomegaly [20, 28, 29]. These studies did not find a significant expansion of HybHPs at 2 days [20] or 2–4 weeks after PHx [28, 30], so the authors assumed that HybHPs respond to a certain type of liver injury. Interestingly, our data showed that Sox9 transiently increased at 3 h and then gradually decreased to the normal level 9 h later. This may explain why no induction of HybHPs was observed in the previous studies at 2 days or even 2–4 weeks post-PHx.

Previous reports have demonstrated hemodynamic changes [31], vagal signaling [32], innate immunity [33], Wnt/β-catenin signaling [34], Notch signaling [35], and Hippo signaling [8] are involved in liver regeneration. Some compounds derived from the gut microbiota could promote liver regeneration by upregulation of inflammation cytokines mediated by TLR4 activation [36–38]. Here, our study further indicated that LPS from the gut microbiota might promote the conversion of hepatocytes into HybHPs by TLR4 activation to accelerate liver regeneration, which demonstrated that the resident microbiota emerged as a key player in liver homeostasis. Studies have revealed the mechanisms involved in the regulation of hepatocyte proliferation, but have not yet illustrated hepatocyte phenotypic plasticity in liver regeneration after PHx. The activation of the NF-κB signaling pathway is essential to induce hepatocytes into the cell cycle after PHx [8, 11, 26]. Accordingly, we demonstrated that the activated NF-κB pathway promotes hepatocyte proliferation. Furthermore, we found that it regulates cell stemness by inducing the upregulation of Oct4, Sox2, and Nanog. More importantly, our results showed for the first time that LPS/NF-κB signaling, apart from regulating proliferation, promotes the conversion of hepatocytes into Sox9^+^HNF4α^+^ cells to participate in liver regeneration after PHx.

Bcl3 is involved in a wide range of biological processes, such as cell survival [39], proliferation [40, 41], immune response [42, 43], inflammation [44], and stemness [22, 45, 46]. Weighted gene coexpression network analysis following PHx indicated that Bcl3 might play a key role in the proliferation stage [47]. Furthermore, Bcl3 might mediate the response of thyroid hormones to liver proliferation [48]. However, the molecular mechanism by which Bcl3 is involved in liver regeneration is not known. Here, we demonstrate that Bcl3 is significantly upregulated and induces the transition of mature hepatocytes into HybHPs after PHx, which is consistent with Bcl3 being required for pluripotency and self-renewal of stem cells [22]. Consistently, Bcl3 was reported to maintain a pathogenic Th1 cell phenotype and prevent conversion to a Th17-like cell with a nonpathogenic phenotype in T cell transfer-induced colitis [12]. Furthermore, previous reports indicated that Bcl3 might participate in the cell cycle and cell differentiation pathways [49], and our study validated the hypothesis. Therefore, Bcl3 might be a new target for promoting liver regeneration by controlling the plasticity and proliferation of hepatocytes.

The Hippo/YAP pathway plays pivotal role in regulating liver growth and cell fate during development, regeneration, and tumorigenesis [5, 20, 50, 51]. Here, our study demonstrates for the first time that Bcl3 mediates the crosstalk between NF-κB and the Hippo/YAP pathway, which further confirms the interplay between Hippo-YAP and NF-κB signaling [52]. Furthermore, Bcl3-induced liver regeneration is mediated by YAP1, which is consistent with the function of YAP1 in chronic liver injury, wherein it regulates hepatocyte phenotypic plasticity and proliferation [5]. We are the first to report that Bcl3 regulates the stability of YAP1 by binding to and inhibiting its ubiquitination. This is consistent with a previous study showing that Bcl3 binds and blocks the ubiquitination of p50 [25]. Additionally, Bcl3 induces the translocation of YAP1 into the nucleus to drive transcription of its target genes, which indicates that Bcl3 functions as a coactivator. However, there is no ubiquitin carboxyl-terminal hydrolase domains in Bcl3, so it will be very interesting to further investigate how Bcl3 inhibits the ubiquitination of YAP1.

Our results demonstrate that Sox9^+^HNF4α^+^ stem-like cells are involved in liver regeneration in the initial phase after PHx and derived from mature hepatocytes as a result of Bcl3 activation. However, it has been reported that overexpressed Bcl3 is closely correlated with a poor prognosis for liver cancer [53]. These results reveal that it is very important to regulate the expression of Bcl3 tightly and specifically to balance regeneration, inflammation, and tumorigenesis. Together, our findings indicate that Bcl3 may be a potential therapeutic target for driving liver regeneration and repair. Additionally, Bcl3 may be a novel therapeutic target in hepatocellular carcinoma.

## Supplementary information

Supplemental Figure legends

Figure S1

Figure S2

Figure S3

Figure S4

Figure S5

Figure S6

Supplemental Table S1

Supplemental Table S2

aj-checklist

Author Contribution Statement

## Data Availability

Correspondence and requests for materials should be addressed to LXW or XRZ.
