# Supplementary material for: LPS/Bcl3/YAP1 signaling promotes Sox9+HNF4α+ hepatocyte-mediated liver regeneration after hepatectomy
Source: Cell Death Dis. 2022 Mar 28;13(3):277. doi: 10.1038/s41419-022-04715-x (PMC8964805; doi:10.1038/s41419-022-04715-x)
Supplement: Supplementary file 1 — Supplemental Figure legends [file 41419_2022_4715_MOESM1_ESM.docx]

**Supplemental Figure Legends**

**Figure S1. Sox9^+^HNF4α^+^ cells are highly proliferative and possess mesenchymal cell characteristics.** (A) A representative picture of triple staining for Sox9 (red), HNF4α (green) and TERT (white) in mouse liver at the indicated times after PHx. (B) Western blotting analysis of liver for TERT expression at the indicated times post PHx (upper) or at 3 hours after removal of the indicated proportion of the liver (bottom). (C) IHC analysis of epithelial cell marker E-cadherin and the mesenchymal cell marker Vimentin in serial liver sections. (D) Representative western blotting analysis of E-cadherin and Vimentin in the liver after PHx. (E) Relative expression of Sox9 revealed by western blotting in pDKD-Control and pDKD-Sox9 shRNA mice at 3 hours after PHx. (F) IHC analysis of Sox9 and Ki67 in serial liver sections. (G) Representative liver images 10 weeks after transplantation of Fah^+^ hepatocytes into *Fah^-/-^* mice. IF analysis of Fah (green) expression was used to evaluate the efficiency of transplantation. (H) Sox9 (red) and Fah (green) double staining in chimeric *Fah^-/-^* mice at hours 0 and 3 after PHx. Nuclei were stained with DAPI. The arrows indicate Sox9^+^Fah^+^ hepatocytes converted from Sox9^-^Fah^+^ hepatocytes.

**Figure S2. The effect of LPS on hepatocytes *in vitro* and *in vivo*.** (A) The relative expression of Sox9 assessed by qRT-PCR in AML12 cells after stimulation with LPS for the indicated times. (B) IF analysis of Sox9 in AML12 cells after administration of LPS for 12 hours. (C) Stem cell and mesenchymal cell markers assessed by qRT-PCR in AML12 cells after treatment with LPS for the indicated times. (D) Representative western blotting analysis of stem cell and mesenchymal cell markers in AML12 cells after treatment with LPS for the indicated times. (E) Representative western blotting analysis of Sox9 in the liver after ligation of a hepatic portal vein branch. (F) Upper: IHC analysis of Sox9 and HNF4α in the hepatic lobe with portal vein ligation or no ligation. Bottom: Quantification of Sox9^+^ and HNF4α^+^ cell numbers. The data are expressed as the mean ± SEM. The measurement results were repeated at least 3 times and the results were similar. *p < 0.05, **p < 0.01, ***p < 0.001, and ****p < 0.0001.

**Figure S3. Bcl3, functioning downstream of the LPS/TLR4 signaling pathway, is involved in liver regeneration after PHx.** (A) Relative expression of Bcl3 assessed by qRT-PCR in AML12 cells after stimulation with LPS for the indicated times. (B) IF analysis of Bcl3 in AML12 cells after administration with LPS for 12 hours. (C) Representative western blotting of Bcl3 in the liver after ligation of a hepatic portal vein branch. (D) Left: Representative Sox9 staining in wild-type and *Bcl3^-/-^* mice after PHx. Right: Quantification of Sox9-positive cell numbers. (E) Representative western blotting analysis of Sox9 in wild-type and *Bcl3^-/-^* mice after PHx. (F) Relative expression of Bcl3, Sox9, and TERT in TLR4-deficient mice after Bcl3 overexpression. (G) IHC analysis of Sox9 in TLR4-deficient mice after Bcl3 overexpression. (H) IHC analysis of PCNA in TLR4-deficient mice after Bcl3 overexpression. The data are expressed as the mean ± SEM. The measurement results were repeated at least 3 times and the results were similar. **p < 0.01, and ***p < 0.001.

**Figure S4.** **YAP1 mediates the positive effect of Bcl3 on liver regeneration after PHx.** (A) Representative western blotting of Sox9 in primary hepatocytes from wild-type or Bcl3-deficient mice after PHx. (B) Predicted location and sequence of mouse κB sites in the promoter of *Sox9*, as identified by the JASPAR-2018 program. (C) ChIP-qPCR was used to analyze whether Bcl3 can bind to the *Sox9* promoter by interacting with κB subunits in AML12 cells. (D) IF analysis of Sox9 (red) and YAP1 (green) in wild-type mouse liver at the indicated times after PHx. (E) Representative western blotting analysis of YAP1 and CTGF in primary hepatocytes from wild-type and Bcl3-deficient mice at the indicated times after PHx. (F) Western blotting was used to analyze YAP1, Sox9 and TERT expression in p-DKD-GFP or p-DKD-shYAP1 mice after PHx. (G-H) IHC analysis of Sox9 and Ki67 in pDKD-GFP or pDKD-shYAP1 mice after PHx. (I) Liver recovery ratio in pDKD-GFP and pDKD- shYAP1 mice at day 3 after PHx. The data are expressed as the mean ± SEM. The three measurement results were repeated 3 times and the results were similar. *p < 0.05, **p < 0.01, and ***p < 0.001.

**Figure S5. YAP1 is activated by the LPS/TLR4 signaling pathway *in vitro* and *in vivo*.** (A-B) Relative expression of YAP1, CTGF, and Cyr61, assessed by qRT-PCR (A) or western blotting (B) in AML12 cells after stimulation with LPS for the indicated times. (C) Western blotting analysis of YAP1 in cytoplasmic and nuclear extracts from AML12 cells after treatment with LPS for the indicated times. (D) Representative western blotting of YAP1 and CTGF in the liver after ligation of a hepatic portal vein branch. The data are expressed as the mean ± SEM. The measurement results were repeated at least 3 times and the results were similar. *p < 0.05.

**Figure S6. Overexpressing YAP1 rescues the inhibitory effect of TLR4 deficiency on liver regeneration after PHx.** (A) Representative western blotting analysis of YAP1 and CTGF in wild-type and TLR4-deficient mice after PHx. (B) The relative expression of YAP1 and Sox9 at the protein level after overexpressing YAP1 in *TLR4^-/-^* mice. (C) IF analysis of Sox9^+^HNF4α^+^ cells after overexpression of YAP1 in *TLR4^-/-^* mice. (D-E) Representative IHC analysis of Sox9 and PCNA in TLR4-deficient mice after YAP1 overexpression. (F) Liver recovery ratio in *TLR4^-/-^* mice treated with pAdeno-EGFP or pAdeno-YAP1 virus. The data are expressed as the mean ± SEM. The three measurement results were repeated 3 times and the results were similar. *p < 0.05, **p < 0.01, and ***p < 0.001.
