# Supplementary figures and images for: LPS/Bcl3/YAP1 signaling promotes Sox9+HNF4α+ hepatocyte-mediated liver regeneration after hepatectomy
Source: Cell Death Dis. 2022 Mar 28;13(3):277. doi: 10.1038/s41419-022-04715-x (PMC8964805; doi:10.1038/s41419-022-04715-x)

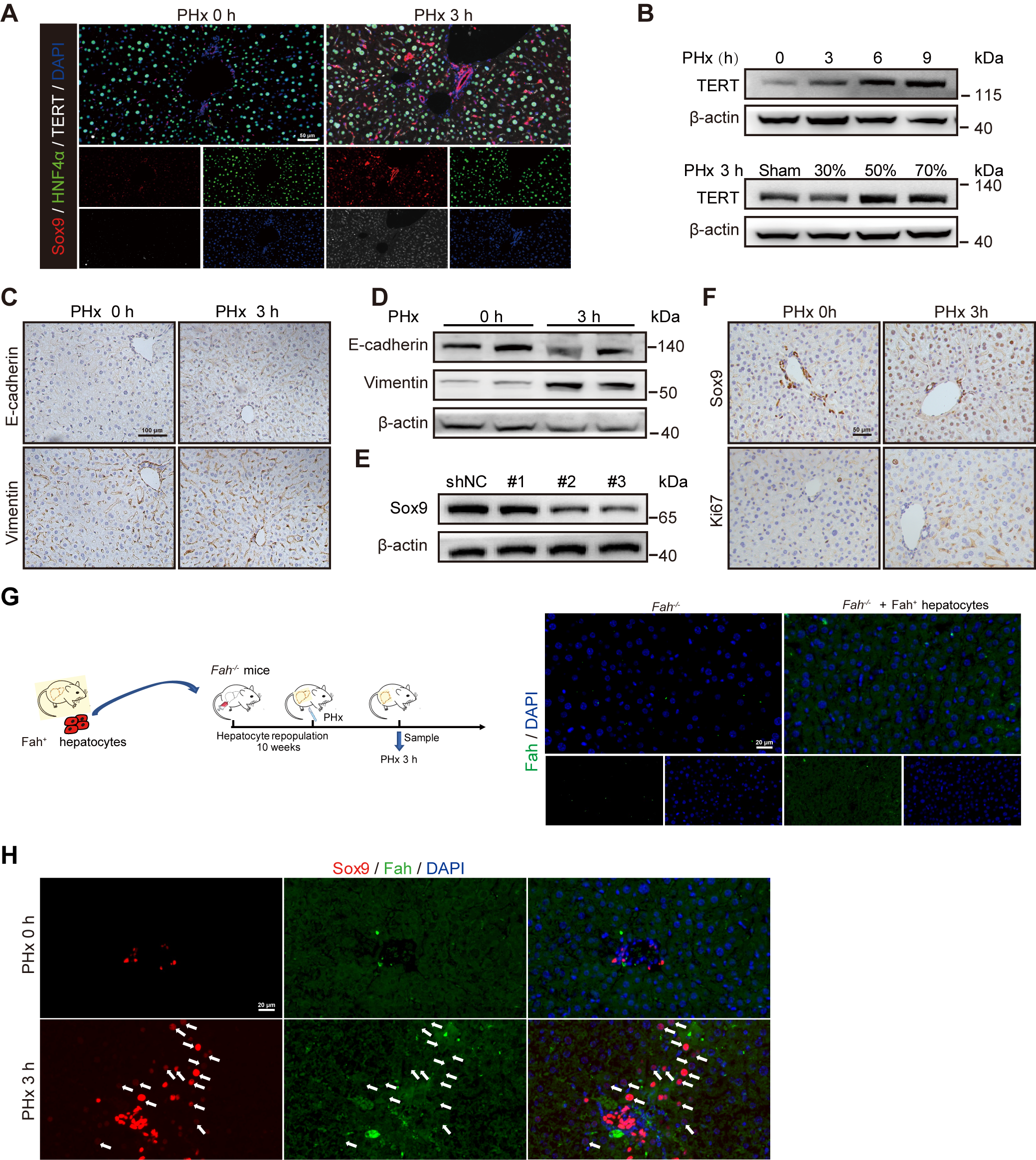

Supplement: Supplementary file 2 — Figure S1 [file 41419_2022_4715_MOESM2_ESM.tif]

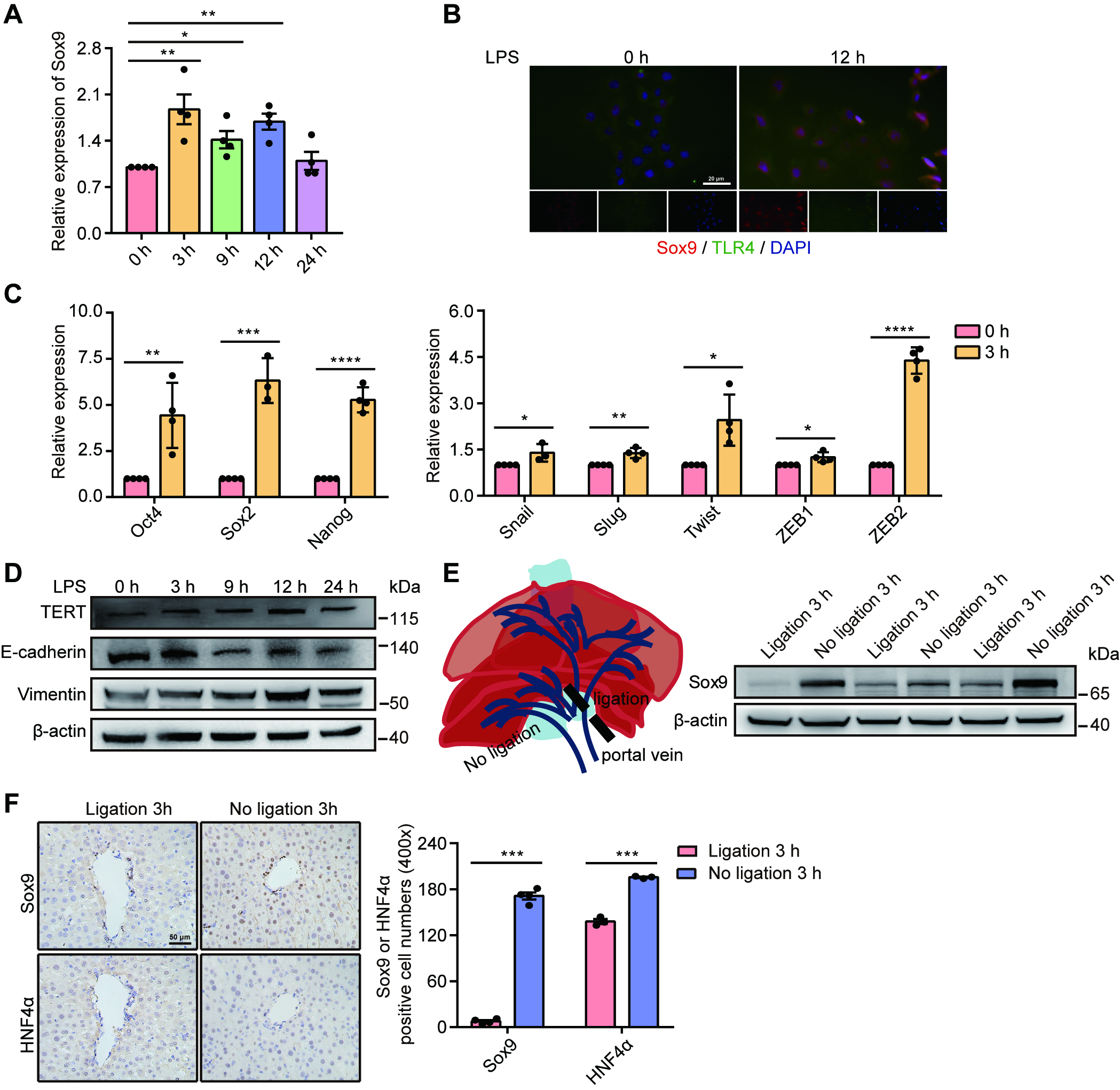

Supplement: Supplementary file 3 — Figure S2 [file 41419_2022_4715_MOESM3_ESM.tif]

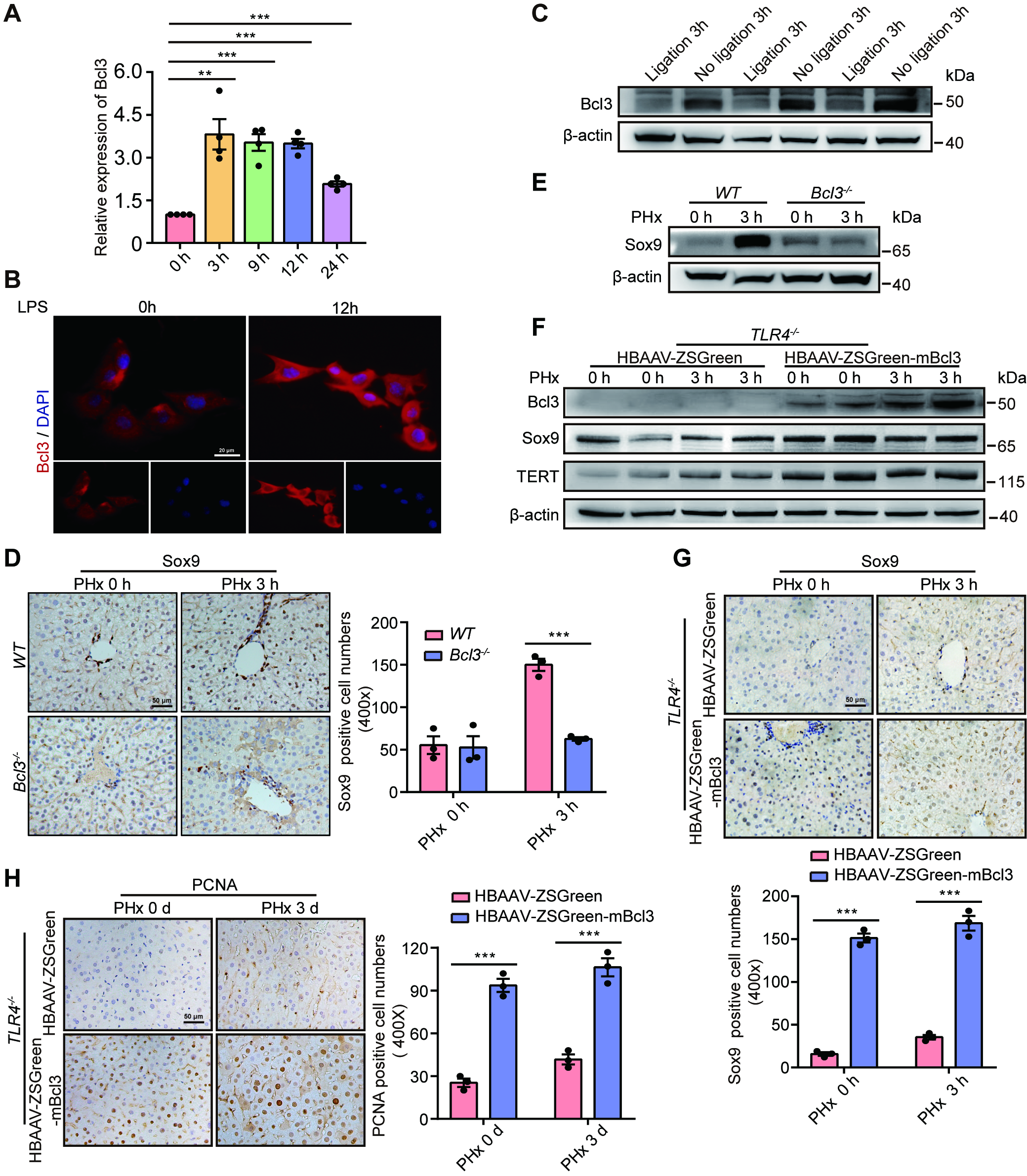

Supplement: Supplementary file 4 — Figure S3 [file 41419_2022_4715_MOESM4_ESM.tif]

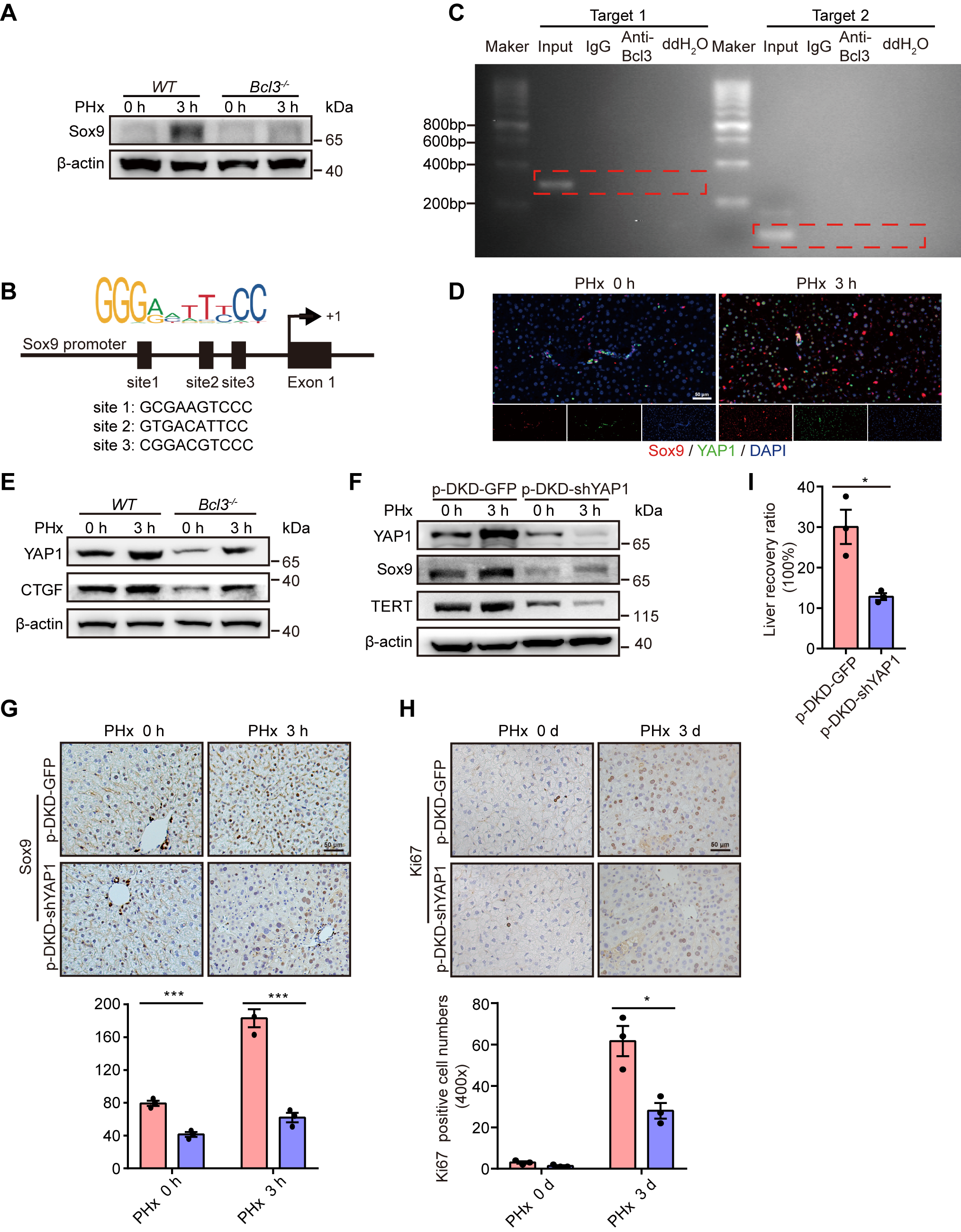

Supplement: Supplementary file 5 — Figure S4 [file 41419_2022_4715_MOESM5_ESM.tif]

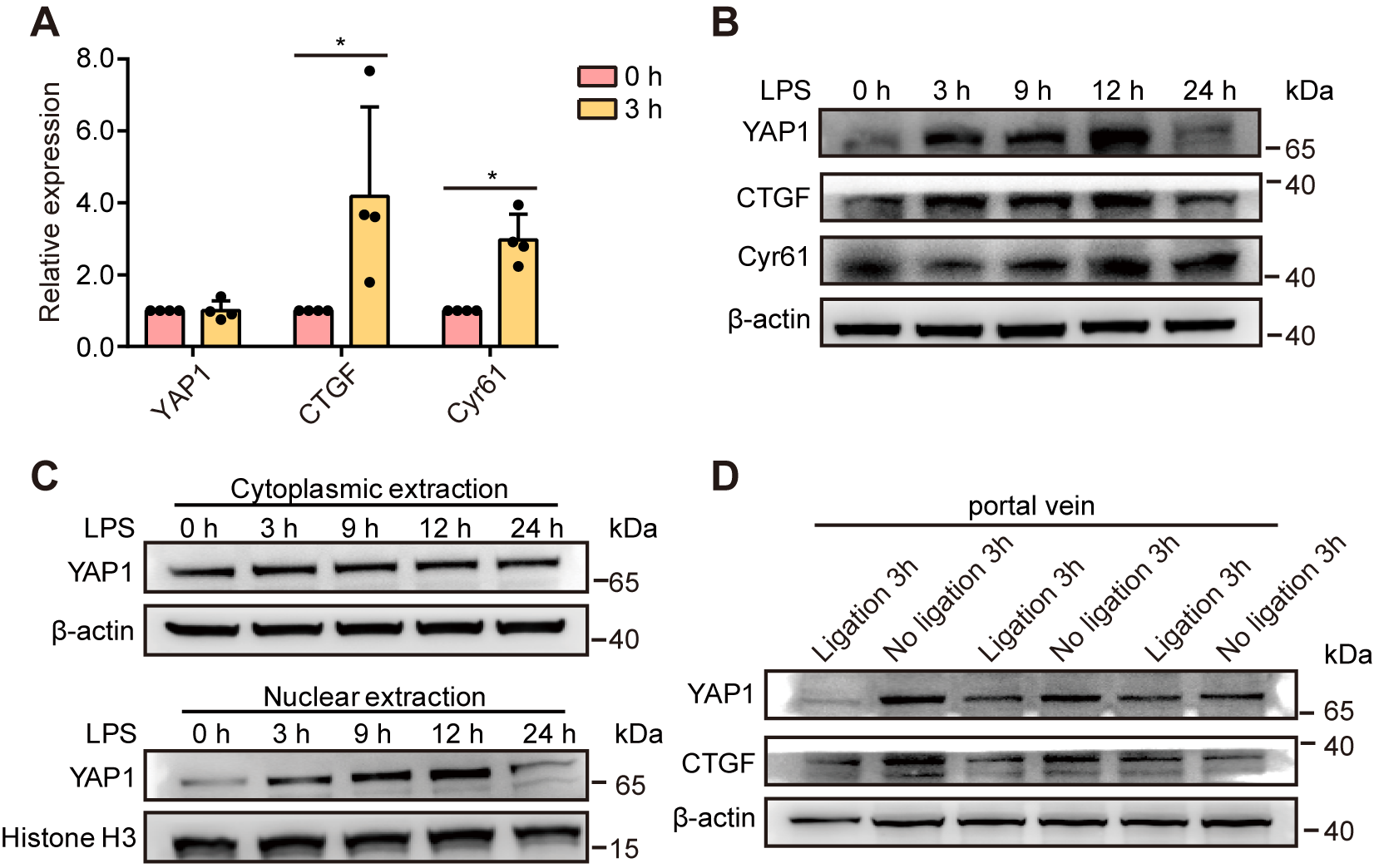

Supplement: Supplementary file 6 — Figure S5 [file 41419_2022_4715_MOESM6_ESM.tif]

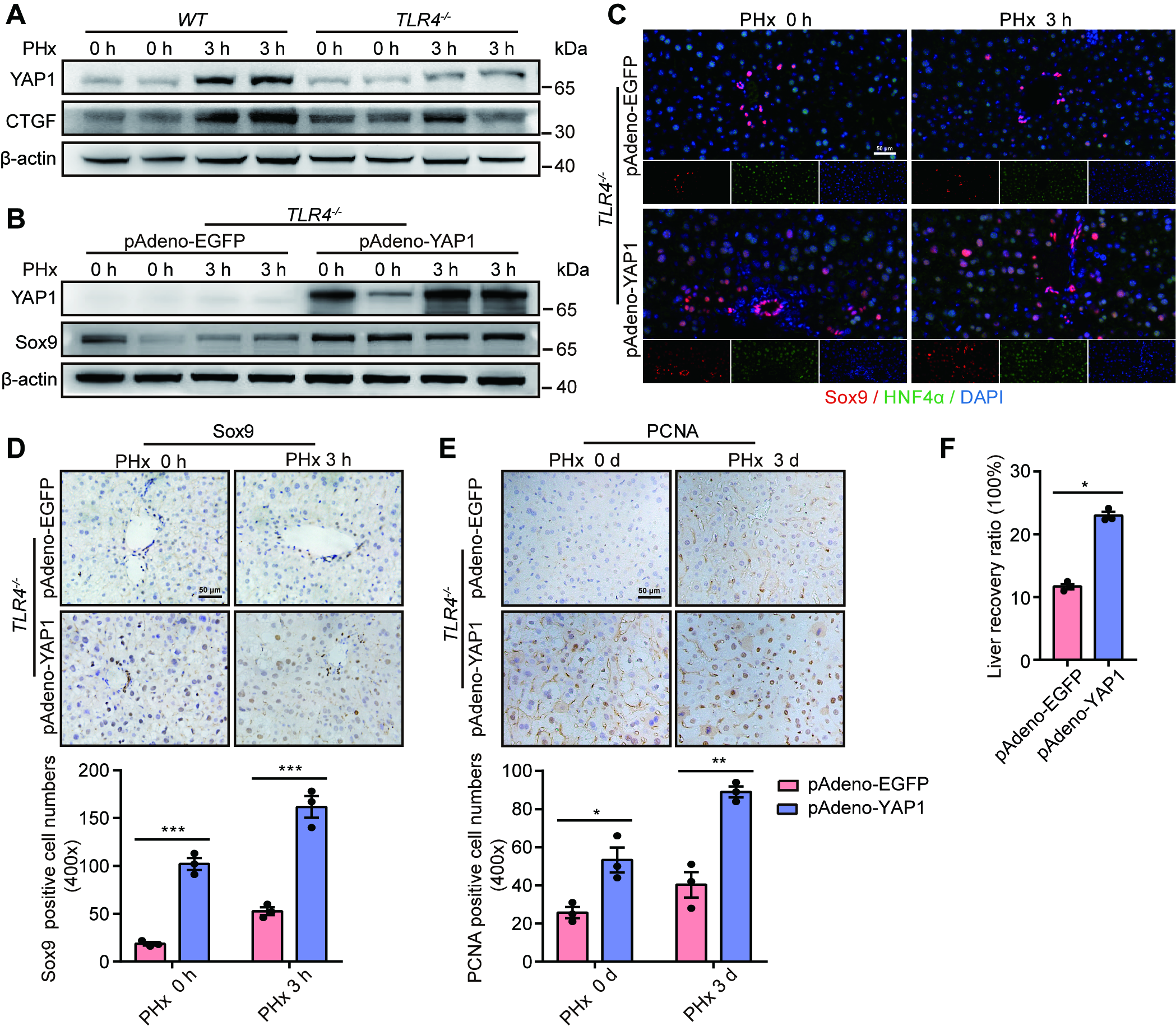

Supplement: Supplementary file 7 — Figure S6 [file 41419_2022_4715_MOESM7_ESM.tif]
