# Supplementary material for: LPS/Bcl3/YAP1 signaling promotes Sox9+HNF4α+ hepatocyte-mediated liver regeneration after hepatectomy
Source: Cell Death Dis. 2022 Mar 28;13(3):277. doi: 10.1038/s41419-022-04715-x (PMC8964805; doi:10.1038/s41419-022-04715-x)
Supplement: Supplementary file 8 — Supplemental Table S1 [file 41419_2022_4715_MOESM8_ESM.docx]

| Primers Names | Sequence 5' to 3' |
| --- | --- |
| Chip-PCR primer |  |
| Target 1 F | TTCCATTGCTGTAAACGCCA |
| Target 1 R | GTCTAGACTAGGACCCTCCACA |
| Target 2 F | GTTGTGGAGGGTCCTAGTCT |
| Target 2 R | CTGGGGAGGATTGTGGCTC |
| Nonspecific target F | GGCCACTGTCGTTTTCGTTG |
| Nonspecific target R | GGGGCCACTGTCAGATGTAT |
|  |  |
| Real-time PCR primer |  |
| Bcl3 F | CACACGCACAAATGTGGTACAC |
| Bcl3 R | ACCACCACTGCCCATCTTATAG |
| YAP1 F | ACCCTCGTTTTGCCATGAAC |
| YAP1 R | TGTGCTGGGATTGATATTCCGTA |
| CTGF F | GGCCTCTTCTGCGATTTCG |
| CTGF R | GCAGCTTGACCCTTCTCGG |
| Cyr61 F | TAAGGTCTGCGCTAAACAACTC |
| Cyr61 R | CAGATCCCTTTCAGAGCGGT |
| Sox9 F | AGTACCCGCATCTGCACAAC |
| Sox9 R | CGAAGGGTCTCTTCTCGCT |
| Oct4 F | TGAGAACCTTCAGGAGATATGCAA |
| Oct4 R | CTCAATGCTAGTTCGCTTTCTCTTC |
| Sox2 F | GGTTACCTCTTCCTCCCACTCCAG |
| Sox2 R | TCACATGTGCGACAGGGGCAG |
| Nanog F | CAGAAAAACCAGTGGTTGAAGACTAG |
| Nanog R | GCAATGGATGCTGGGATACTC |
| E-cadherin F | CAGTTCCGAGGTCTACACCTT |
| E-cadherin R | TGAATCGGGAGTCTTCCGAAAA |
| Vimentin F | CGTCCACACGCACCTACAG |
| Vimentin R | GGGGGATGAGGAATAGAGGCT |
| Snail F | CACACGCTGCCTTGTGTCT |
| Snail R | GGTCAGCAAAAGCACGGTT |
| Slug F | CAGCGAACTGGACACACACA |
| Slug R | ATAGGGCTGTATGCTCCCGAG |
| Twist1 F | GGACAAGCTGAGCAAGATTCA |
| Twist1 R | CGGAGAAGGCGTAGCTGAG |
| ZEB1 F | ACCGCCGTCATTTATCCTGAG |
| ZEB1 R | CATCTGGTGTTCCGTTTTCATCA |
| ZEB2 F | AAACGTGGTGAACTATGACAACG |
| ZEB2 R | CTTGCAGAATCTCGCCACTG |
| β-actin F | GTGACGTTGACATCCGTAAAGA |
| β-actin R | GCCGGACTCATCGTACTCC |

**Supplemental Table S1: Chip-PCR and Real-time PCR primers**
