# Supplementary material for: LPS/Bcl3/YAP1 signaling promotes Sox9+HNF4α+ hepatocyte-mediated liver regeneration after hepatectomy
Source: Cell Death Dis. 2022 Mar 28;13(3):277. doi: 10.1038/s41419-022-04715-x (PMC8964805; doi:10.1038/s41419-022-04715-x)
Supplement: Supplementary file 9 — Supplemental Table S2 [file 41419_2022_4715_MOESM9_ESM.docx]

| **Supplemental Table S2: siRNA target sequences** | |
| --- | --- |
| targets | Sequence 5' to 3' |
| pDKD-CMV-eGFP-U6-shRNA control | TTCTCCGAACGTGTCACGT |
| pDKD-CMV-eGFP-U7-shRNA Sox9 #1 | GCAAGAACAAGCCACACGT |
| pDKD-CMV-eGFP-U7-shRNA Sox9 #2 | GGAGGAAGTCGGTGAAGAA |
| pDKD-CMV-eGFP-U7-shRNA Sox9 #3 | CCACGGAACAGACTCACAT |
|  |  |
| pDKD-CMV-eGFP-U7-shRNA YAP1 | AAGCGCTGAGTTCCGAAATCT |
|  |  |
| pLDK-CMV-EGFP-2A-Puro-U6-shRNA control | TTCTCCGAACGTGTCACGT |
| pLDK-CMV-EGFP-2A-Puro-U7-shRNA Bcl3 #315 | CCAGGGACCTTTGATGCCCATTTAC |
| pLDK-CMV-EGFP-2A-Puro-U7-shRNA Bcl3 #764 | CCTGGAGGTTCGCAATTAT |
