# Supplementary material for: LPS/Bcl3/YAP1 signaling promotes Sox9+HNF4α+ hepatocyte-mediated liver regeneration after hepatectomy
Source: Cell Death Dis. 2022 Mar 28;13(3):277. doi: 10.1038/s41419-022-04715-x (PMC8964805; doi:10.1038/s41419-022-04715-x)
Supplement: Supplementary file 11 — Author Contribution Statement [file 41419_2022_4715_MOESM11_ESM.docx]

**Author contributions**

LXW and XRZ contributed to the design and supervision of the study; CCS, YYJ and SMZ were responsible for conducting the experiments, acquisition and analysis of data, and manuscript writing; XY, YMH, YM, YHH and FY conducted experiments; and LG, WTL, DDS and RL analyzed and interpreted the data. LXW, XRZ, CCS, YYJ, SMZ, XY, YMH, YM, YHH, FY, LG, WTL, DDS and RL all approved the final version for publication.
